# Leishmaniasis in Saudi Arabia: Current situation and future perspectives

**DOI:** 10.12669/pjms.36.4.2121

**Published:** 2020

**Authors:** Elfadil Abass, Zainab Al-Hashem, Lamya Zohair Yamani

**Affiliations:** 1 Elfadil Abass, Department of Clinical Laboratory Science, College of Applied Medical Sciences, Imam Abdulrahman Bin Faisal University, Dammam, Saudi Arabia; 2 Zainab Al-Hashem, Department of Clinical Laboratory Science, College of Applied Medical Sciences, Imam Abdulrahman Bin Faisal University, Dammam, Saudi Arabia; 3 Lamya Yamani, Department of Clinical Laboratory Science, College of Applied Medical Sciences, Imam Abdulrahman Bin Faisal University, Dammam, Saudi Arabia

**Keywords:** Epidemiology, Leishmaniasis, Saudi Arabia

## Abstract

**Background and Objective:**

Leishmaniasis is endemic in Saudi Arabia with cases reported in many regions. This review refers to publications on leishmaniasis in Saudi Arabia and discusses issues related to parasite species, clinical manifestation and diagnosis.

**Methods:**

This research was done at Imam Abdulrahman Bin Faisal University, Dammam, Saudi Arabia by systematic literature search on PubMed and Google Scholar databases from 1989 to 2018. Selection criteria included original articles reporting on visceral leishmaniasis (VL) or cutaneous leishmaniasis (CL) in Saudi Arabia.

**Results:**

The search identified 16 eligible articles, six for VL and 10 for CL. VL was reported in areas known to be non-endemic. *Leishmania donovani* was the main cause for human VL while *Leishmania infantum* seemed to cause the disease in animals. Dogs were considered the main reservoir hosts and black rats (*Rattus rattus*) were potential hosts. VL mainly affected infants and young children. It is important to note that VL diagnosis was based on either invasive parasite detection procedures or serologically using indirect hemagglutination test. CL represented the most frequent clinical form with the main endemic foci reported in the South-West and Eastern regions. CL appeared to have no demographic or socioeconomic restriction; it affected both rural and urban citizens, with the majority occurring among farmers. Travelling was recognized as an important risk factor. *Leishmania tropica* and *Leishmania major* were recognized as the main causes for CL.

**Conclusion:**

This report summarizes the potential risks for VL and CL in Saudi Arabia in areas known to be non-endemic. There are substantial gaps in knowledge and practices in regard to leishmaniasis in Saudi Arabia, highlighting the need for more research and medical surveillance targeting the disease in humans and animals.

## INTRODUCTION

Leishmaniasis is a vector-borne disease caused by different species belonging to the genus *Leishmania*. These species cause various clinical manifestations ranging in severity from self-limited cutaneous lesions to life-threatening visceral disease, including cutaneous leishmaniasis (CL), visceral leishmaniasis (VL), mucocutaneous leishmaniasis (MCL), and post kala-azar dermal leishmaniasis, PKDL.^1^ The disease is categorized into two types: zoonotic leishmaniasis (where wild and domestic animals are considered main reservoir hosts) and anthroponotic leishmaniasis (where humans are the source of the infection). Several domestic and wild mammalian hosts are involved in the transmission cycle of the disease, including certain rodents and dogs.^1^,^2^

VL is the most severe form of the disease. It is caused by various species of *Leishmania* in different endemic regions; *L. donovani* in Asia and East Africa and *L. infantum* in the Mediterranean area, Middle East, central Asia and America. It is the most severe form of the disease and still associated with high mortality.^1^ Laboratory findings include anemia, leukopenia, thrombocytopenia, hypoalbuminemia, and hypergammaglobulinemia. Kidney damage in VL is well-known and can appear as glomerulonephritis, acute or chronic renal disease.^3^,^4^

The VL suspicion is present if fever persists more than two weeks in the presence of splenomegaly in individuals living in or having visited known VL-endemic areas. Clinical diagnosis is confirmed by various laboratory methods. These methods are based on detection of the parasite in aspirates collected from spleen, liver, bone marrow or lymph nodes. Sensitivity of these methods depends on the type of sample, being less sensitive for lymph nodes while more sensitive for spleen samples.^2^,^5^ Due to the invasive nature of sample collection and low sensitivity, new methodologies based on detection of specific *Leishmania* antibody are currently used. The direct agglutination test (DAT) using intact promastigote antigen and enzyme-linked immunosorbent assay (ELISA) using refined recombinant proteins of *Leishmania* are most common methods. These methods are used for detection of VL in humans and animal hosts with variable diagnostic accuracy in the various endemic regions.^6^

Worldwide, CL is the most common form of leishmaniasis, causing the greatest disease burden. It occurs across the Indian subcontinent, through the Mediterranean region and from Africa to America. Most of the cases occur in six countries including Afghanistan, Brazil, Colombia, Iran, Algeria and Syria.^7^,^8^

CL is a self-limiting skin disease, causing skin ulcers on the uncovered body parts at the place of the infected sand fly vector bite. The appearance of characteristic lesions in areas with high endemicity of CL is enough to establish the clinical diagnosis. However, laboratory tests are required to distinguish leishmaniasis from several other skin diseases. The diagnosis is classically based on direct detection of *Leishmania* in lesion smears stained by Giemsa-stain or by culture.^9^,^10^ PCR techniques are highly sensitive and help to determine the parasite species, but it requires painful and invasive procedures for sample collection.^10^,^11^ Serological diagnosis is frequently used in epidemiological studies of leishmaniasis. It is an easy and quick approach, but its sensitivity is low due to limited circulating antibodies and potential antigen diversity of parasites that cause the disease.^12^,^13^

## METHODS

### Literature Search Strategy

This systematic literature search was done at Imam Abdulrahman Bin Faisal University, Dammam, Saudi Arabia. It was conducted by a literature search identifying epidemiological studies reporting leishmaniasis in Saudi Arabia. The search was performed on electronic databases using the PubMed and Google Scholar. The search was performed from 1989-2018. Keywords that were used included: epidemiology, leishmaniasis and Saudi Arabia. Only original research articles written in English language were selected. Furthermore, a Google search was used as an additional source of data.^14^ These articles were also included in the data analysis.

## RESULTS

**Fig.1:**
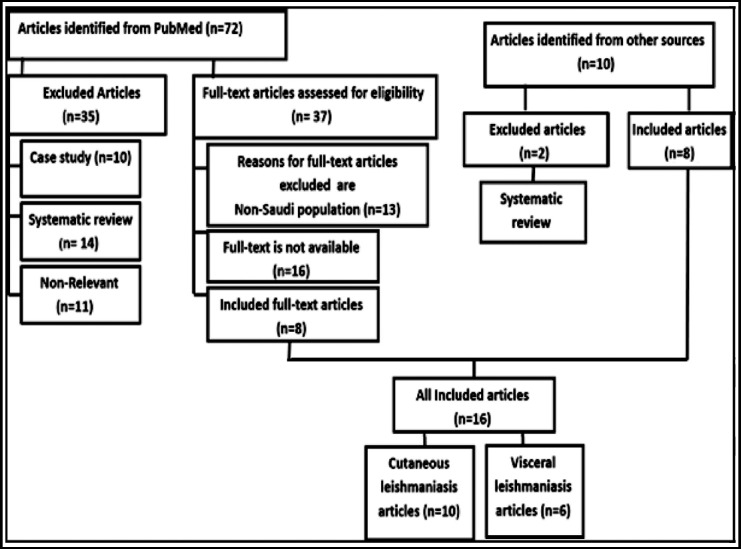
Flow chart of included and excluded scientific articles.

Sixteen eligible articles were identified (Fig.1), six studies reported VL and 10 articles reported CL. Results showed an uneven distribution of both diseases in the Kingdom. It was not possible to count the total number of individuals infected by the disease. In most of the published studies, only selected cases were involved (Table I and II). None of these studies reported outbreaks or co-infections with other diseases. There were no reports on parasite characterization and genetic heterogeneity. The role of black rats in epidemiology of the disease was not confirmed. Also, there were no reports in regard to usage of new laboratory tests for the disease confirmation. The published studies also lack information on the use of new leishmaniasis drugs/regimens.

**Table-I:** Included studies of VL in Saudi Arabia.

Author, Year [Reference]	Study Year	Study area	Total Identified Cases	Diagnosis Method
Mokhtar et al. (2017)^15^	NA	Najran, South West	32	IHAT
Jamil.et.al. (2012)^16^	1985-2007	Abha, South West	123	Parasite detection in bone marrow
Jamil.et.al. (2012)^17^	1985-2008	Aseer, South West	582	Parasite detection in bone marrow, IHAT
Jack et al. (2005)^18^	1988	Al Madinah, West	7	Parasite detection in bone marrow, IHAT
Al-Orainey et al. (1994)^19^	NA	Gizan, South West	121	Parasite detection in liver/spleen, culture, IHAT
Ibrahim.et al. (1992)^20^	1989-1990	Gizan, South West	NA	Parasite detection in liver/spleen, culture

**IHAT:** indirect hemagglutination assay test; **NA:** no available data.

**Table-II:** Included Studies of CL in Saudi Arabia.

Author, Year [Reference]	Study Year	Study area	Total Identified Cases	Diagnosis Method
Hawash et al. (2018)^21^	2016-2017	Al-Taif, West	90	Parasite detection in skin smears, OligoC- PCR, and kDNA PCR
Alahmad et al. (2018)^22^	2009-2010	Al-Hassa, East	901	Parasite detection in skin smears
Haouas et al. (2017)^23^	2015-2017	Hail, North West	37	Parasite detection in skin smears and SSU-rDNA- PCR
Barradah (2017)^24^	2007-2016	Majmaah, Riyadh Province	87	Parasite detection in skin smears
Alanazi et al. (2016)^25^	2009-2013	Dawadim, Riyadh Province	370	Parasite detection in skin smears
Faraj& Lake (2014)^26^	1996-2007	Aseer, South West	NA	Parasite detection in skin smears
El-Beshbishy et al. (2013)^27^	2010-1012	Al-Madinah, West	34	kDNA PCR
Uthman et al. (2005)^28^	1997-1999	Al-Khobar, East	120	Parasite detection in skin smears
Al-zahrani et al. (1989)^29^	1987	Al-Baha, Aseer, West	2302	Parasite detection in skin smears
Dye et al. (1989)^30^	1986	Al-Hassa, East	284*	Identification of active lesions and leishmanial scars

**kDNA PCR:** kinetoplast DNA polymerase chain reaction, **SSU-rDNA PCR:** small subunit ribosomal DNA PCR.

* The number included cases with active lesions (117) and leishmanial scars (n=167).

### Visceral leishmaniasis

### Epidemiological Characteristics

The major endemic areas that reported VL in Saudi Arabia were in the South and South-West. Sporadic cases were also reported in the city of Al-Madinah. Endemic areas included Gizan, Najran and Asser. In these areas, VL was characterized by seasonal variation with emergence of many cases in late spring and summer and few cases were observed in the winter. In Gizan, VL was confirmed in both humans and animals. *L. donovani* LON42 was associated with human infections while both *L*. *donovani* LON42 and *L. infantum* LON 49 were isolated from black rats (*Rattus rattus*). *L. infantum* LON49 was isolated only from dogs. In Najran, other animals might act as reservoirs for the parasite, such as goats and sheeps.

Most of the published articles reported VL among infant and adolescence in Gizan, Aseer and Al-Madinah. There was no gender restriction; both male and females were equally affected. In a study carried out in Najran involving 384 human samples, anti-*Leishmania* antibodies were significantly higher among old individuals.

### Clinical Features

The common clinical features of VL among pediatric patients included fever (100%), pallor (>95%), hepatosplenomegaly (>90%) and lymphadenopathy (>90%). Other clinical presentations included abdominal distention, anorexia, and weight loss. However, none of the patients showed skin lesions (PKDL) while lymphadenopathy was a less common clinical finding. VL patients showed also abnormal laboratory results including anemia, leukopenia, thrombocytopenia, hypoalbuminemia and hypergammaglobulinemia. Abnormalities in liver functions were also observed.

### Diagnostic Features

In endemic areas, primary VL suspected cases and diagnosis was based on clinical signs and symptoms. Laboratory diagnosis was based on either direct detection of *Leishmania* in bone marrow aspirates or serologically using indirect hemagglutination test (IHAT) kit (Siemens Healthcare Diagnostic, Marburg, Germany). IHAT detects anti-*Leishmania* antibodies in patients’ sera with hemagglutination cutoff titers of 1: 32-1: 64. This test performed very well with high sensitivity and specificity. Sensitivity of bone marrow aspiration for detection of *Leishmania* was also high (92.7%). None of the studies detected *Leishmania* in lymph node aspirates. Some studies used splenic and liver aspirates and isolation of the parasite in diphasic media for confirmation of the disease.

### Cutaneous leishmaniasis

### Epidemiological Characteristics

CL was endemic in various parts of Saudi Arabia, with most of the cases reported in East, West, South-West, North-West and the Southern regions. Endemic areas included Al-Hassa, Aseer, Al-Baha, Al-Madinah, Al-Taif and Hail. Several cases were also reported in areas known to be non-endemic such as Al-Khobar and Riyadh province. CL was more prevalent among male than female. Infection included all ages with infant and young adults being the majority affected. There was no socioeconomic restriction for the disease. The affected populations included both rural and urban citizens, mainly among farmers. Temperature was recognized as an important factor associated with CL transmission in Aseer. Travelling was also recognized as a risk factor. Studies confirmed *L. tropica* and *L. major* as the causative agents for the disease in Saudi Arabia. Various zymodemes of *L. tropica* were identified in the South-West region, including LON-71, LON-72, LON-73, LON-10 and LON-63. *L. major* zymodeme LON-1 and LON-4 were also reported in the region.

### Clinical Features

CL manifested as skin lesions on the face and exposed parts of the body. The number of skin lesions found in each patient differs, with the majority developing 2–3 lesions. In many cases the lesions were excoriated and were observed as insect bites. Ulcerative lesions varied in size (from 2-10 cm in diameter), with moderately rolled edges and granulomatous bases. The lesions were painful when associated with secondary bacterial infection and inflammation.

### Diagnostic Features

Parasite culture and microscopic examination of smears were the most common laboratory methods for CL diagnosis. These methods were recommended to be used together for greater efficacy. Molecular tools such as PCR based on ITS1 and kDNA was used to facilitate the diagnosis and identification of the parasite. These methods found to be more sensitive than parasite culture and microscopic examination. They detected most of CL cases that were negative by direct examination methods. In a single study, kDNA PCR proved to be more sensitive than microscopy and OligoC-TesT PCR.

## DISCUSSION

This study reports epidemiological data on the most common forms of leishmaniasis in Saudi Arabia. Although Saudi Arabia has experienced high prevalence of leishmaniasis, only minimal recent data has been published on this disease. Reporting updated information is crucial for planning any effective strategy for disease control. These strategies require detection of the disease in humans and animals, identification of reservoir hosts and sand fly vectors.^31^ The general department of the ministry of health in Saudi Arabia controls vector-borne and communicable disease has launched national policy for control of the disease in the country with a case notification system. This has led to marked reduction in the incidence of CL.^32^

In Saudi Arabia, most of the studies passively detected leishmaniasis with varying incidence rates in the different regions and among individuals that were originated from other countries. Estimation of the disease burden on basis of passive reporting would likely underestimate the magnitude of this disease, rendering control programs ineffective. These programs would have achieved better results through active case detection, through which asymptomatic or cases with atypical clinical presentations can be identified. Asymptomatic *Leishmania* infections are not well defined but are often serologically identified. Such asymptomatic infected hosts may serve as reservoirs, transmitting the disease silently. These reported cases are considered to be important reservoir hosts of the parasite, presenting an additional challenge in assessing *Leishmania* infection. It is important to note that asymptomatic *Leishmania* carrier individuals posses decreased antibody titers as compared to clinical VL cases.^33^ Indeed, seropositive individuals have increased chance to progress to symptomatic VL cases. However, substantial numbers of VL seropositive patients will never develop a clinical disease. In reality, it is hard to judge whether an apparently healthy seropositive individual is truly infected or not.

In recent years, important changes in epidemiology of leishmaniasis have been noticed. In addition to rural areas, leishmaniasis has become common in urban settings. Classically, VL affects adults that are more exposed to sand fly vectors. This classical picture has changed; the disease became more prevalent among infants and young children.^34^ This seems to be the case in Saudi Arabia, where VL has been reported in younger age groups. Indeed, exposure to *Leishmania* may lead to development of protective immunity. This could be the reason of the high prevalence of VL among infant and young children. It has been shown that, VL is more common among men than women due to the nature of their work. In Saudi Arabia, women have a special situation. They often stay indoors or cover their body when outdoor, leading to less exposure and thus less susceptibility to *Leishmania*.

Diagnosis of VL can be challenging due to the overlap of VL and other disease areas such as malaria, typhoid, tuberculosis, and HIV. These diseases can coincide with VL. Population movement and increased travelling have led to appearance of VL in areas that were previously free of the disease. In such cases, the patients may develop atypical clinical presentation and may remain undetected. Recently, Alwazzeh and Alhashimalsayed have reported a case of a 42-years old patient in Al-Khobar (Eastern province), a known non-endemic VL area.^4^ This patient was originally from Gizan, but VL was not considered at first check-up due to atypical clinical presentation. After three months’ delay, VL was diagnosed serologically at the Mayo Clinic Labs. This case could have been diagnosed earlier if serological tests were available.

CL has a wide distribution across the world, reaching from Asia, through Middle East and North Africa, to North-and South America. Saudi Arabia has the fourth highest prevalence rate of zoonotic CL after Afghanistan, Iran and Pakistan. It has been shown that factors such as poor access to health facilities along with the moderate nature of the disease (self-healing ulcers) have contributed to the poor passive reporting of the cases.^35^ Therefore, the actual prevalence and incidence of the disease would be higher than reported previously. Also, accurate diagnostic test systems would help identification of these cases.

Molecular tools have identified *L. major* and *L. tropica* as causative agents of CL in Saudi Arabia, with the majority of the cases caused by *L. major*. *L. tropica* seems to be less prevalent, occurring within small foci in the west and south-west regions.^23^,^27^ Despite the high prevalence of the disease in the country, few studies have been focused on identification of *Leishmania* species and little is known about molecular and isoenzyme characterization of the parasites species. This review identified poor application of control tools, including unavailability of modern diagnostics and treatment regimens. In most of the previous studies, diagnosis was based on clinical examination and passive detection of the parasite in skin biopsies. Indeed, many skin conditions may display similar clinical features resembling CL, thus laboratory diagnosis is essential. Additionally, considering the high toxicity and treatment cost, it is unacceptable to treat unconfirmed cases i.e., expose them to the medication.

## CONCLUSION

This report highlights potential risks for VL and CL in Saudi Arabia in areas known to be non-endemic. There are also crucial gaps about diversity of the reservoir host and their role in the transmission cycle of *Leishmania*. We recommend active case detection using new test systems to identify the real number of undetected cases and to evaluate the magnitude of the problem.

### Authors’ Contribution

**EA**, conceptualization, design, interpretation of data, project administration, supervision, writing and editing.

**ZA**, methodology, writing manuscript draft.

**LZY,** checked validity of data, revise and editing of manuscript.
